# Auriculotherapy in primary care: profile of graduates from a large-scale course

**DOI:** 10.11606/s1518-8787.2025059006715

**Published:** 2025-10-24

**Authors:** Lúcio José Botelho, Charles Dalcanale Tesser, Melissa Costa Santos, Emiliana Domingues Cunha da Silva, Ari Ojeda Ocampo Moré, Fátima Terezinha Pelachini Farias, Lilian Elizabeth Diesel

**Affiliations:** I Universidade Federal de Santa Catarina Centro de Ciências da Saúde Departamento de Saúde Pública Florianópolis SC Brazil Universidade Federal de Santa Catarina. Centro de Ciências da Saúde. Departamento de Saúde Pública. Florianópolis, SC, Brazil; II Prefeitura Municipal de Florianópolis Secretaria de Saúde de Florianópolis Florianópolis SC Brazil Prefeitura Municipal de Florianópolis. Secretaria de Saúde de Florianópolis. Florianópolis, SC, Brazil

**Keywords:** Complementary Therapies, Auriculotherapy, Primary Health Care, Continuing Education

## Abstract

**OBJECTIVES:**

To present the profile of graduates from a blended learning course in auriculotherapy and their perception of user acceptance and clinical outcomes of this integrative and complementary health practice, currently the most widely implemented in the Brazilian Unified Health System. The course is offered free of charge to primary health care professionals.

**METHOD:**

A digital questionnaire was sent in 2023 by email to all graduates to date (n = 13,581), addressing their professional profile, their perception of user acceptance and the clinical outcomes of auriculotherapy. Data were analyzed using descriptive statistics.

**RESULTS:**

A total of 5,461 professionals responded (41.34% of graduates from nine editions of the course, each with multiple classes of around 50 to 100 students). Most were women (87.82%), nurses (33.55%), physical therapists (11.59%), psychologists (5.40%), pharmacists (9.65%), nutritionists (8.05%), dentists (7.57%), and physicians (5.57%); aged 30 to 49 years (77.7%); working in primary health care (80.44%); without prior experience in integrative and complementary health practice (73.58%). After the course, 56.31% reported practicing auriculotherapy. User acceptance was reported as high or very high by 73.6% and as moderate by 22.14% of practitioners. Clinical outcomes were reported as very good or good by 79.72% and as moderate by 18.35%. of practitioners.

**CONCLUSION:**

The graduates of the auriculotherapy course who responded to the survey are mostly women, family health professionals and members of multi-professional teams working in primary health care, who report higher user acceptance and perceive good clinical outcomes from the use of auriculotherapy.

## INTRODUCTION

A few decades ago, the previous trend of disregarding non-scientific (traditional or imported) health care practices was reversed in scientific discussion and in health systems^1^, mostly referring to them as traditional, complementary, or integrative medicines^2^. Since the 1990s, their consistent demand by large sections of the population in high-income countries has been recognized^3^, with growing inclusion in health systems^2^ and an increase in scientific research on the subject^4^.

In Brazil, the *Política Nacional de Práticas Integrativas e Complementares* (PNPIC – National Policy for Integrative and Complementary Practices) was launched in 2006^5^. Since then, the offer of integrative and complementary health practices (ICHP) has been growing, especially in primary health care (PHC)^6^. In 2017 and 2018, the diversity of ICHP in the Unified Health System (SUS) was expanded to 29 modalities^7,8^.

The clinical use of ICHP is predominant in the Brazilian private sector^9^. Despite their growth in the SUS and the PNPIC’s directive for permanent education on the subject, the latter is still incipient, and few initiatives train professionals in the clinical practice of any ICHP. One such initiative is the blended learning course in auriculotherapy, funded by the Brazilian Ministry of Health and run by the Universidade Federal de Santa Catarina (UFSC).

Auriculotherapy is a complementary care technique associated with traditional Chinese medicine (TCM) that consists of punctual physical stimuli in the auricle to treat health problems, most commonly using needles (auricular acupuncture) or spherical plant seeds adhered to the skin, which is the method taught in the aforementioned course. There has been significant development of scientific models of auriculotherapy’s mechanisms of action^10^, supporting the plausibility of its effectiveness.

This course has been considered responsible for the growth of auriculotherapy in Brazilian PHC^11^, making it the most widely offered ICHP in the SUS^12^. There is a clear (almost linear) association between the increase in the number of graduates from the course and the increase in the production of auriculotherapy sessions in PHC, as observed in the SUS information systems (Graph).

**Graph:**
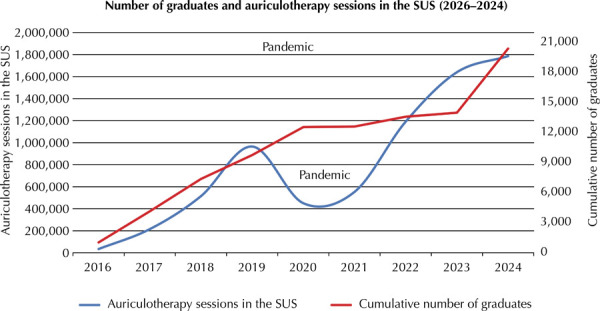
Course graduates and auriculotherapy sessions in the Brazilian Unified Health System.

This course is the world’s largest initiative to train conventional professionals in a complementary therapy in PHC^13^. By the end of 2024, approximately 20,000 SUS professionals working in almost half (46%) of Brazilian municipalities had been trained (Figure).

**Figure:**
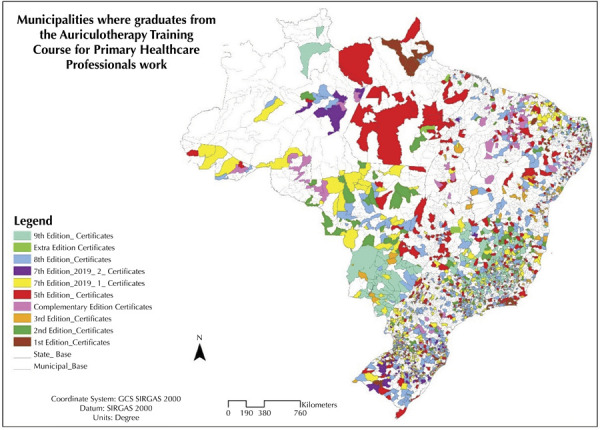
Municipalities where graduates were working at the time of the course.

Local studies and scientific evidence suggest that auriculotherapy is widely accepted by professionals and users for problems of high prevalence and relevance in PHC, such as mental health problems^14^and musculoskeletal pain, especially chronic pain, which affects approximately 30% of the population^15^.

This article seeks to present the profile of the course graduates, their work with auriculotherapy and their perception of user acceptance and the clinical outcomes of this technique, as well as discussing the potential for its widespread clinical use among PHC professionals. Before the research method and results, the course is briefly described.

### About the Course

The initiative to create the auriculotherapy course came from the Brazilian Ministry of Health, which commissioned it from UFSC at the end of 2014. In 2015, the teaching material was prepared by a multidisciplinary team from UFSC and the Florianópolis Municipal Health Department, which had previous experience with in-person auriculot herapy courses.

The course structure was developed based on the World Health Organization (WHO) guidelines for TCM training, but without adhering to the internationally standardized auricular acupuncture nomenclature and auricular map. Due to its introductory nature and the need for a teaching approach adapted to PHC, the course integrated the three main rationales of contemporary auriculotherapy: TCM, auricular reflexology, and scientifically known neurophysiological mechanisms. The pedagogical model created incorporated the auricular points most commonly used in clinical practice and sought an accessible approach based on both scientific literature and traditional TCM references^13^.

A pilot course was held in 2016 whose audiovisual material was severely criticized and reformatted into recorded video lessons in early 2017. Since then, it has been offered in successive editions to senior PHC professionals.

The course consists of five sequential, self-instructional distance learning modules installed on the Moodle digital platform, each with a handout and two to three video lessons, scheduled to be taken over six weeks: 1) introduction to ICHP; 2) ear reflexology; 3) auriculotherapy in the TCM logic; 4) auriculotherapy in the light of biomedicine; and 5) use of auriculotherapy in PHC. Students take an assessment and, if they pass, move on to the next module (they can repeat the assessment as many times as necessary until they pass).

In addition to the handouts and video lessons, an auricular points map (in digital format and as a laminated print) and an online resource for studying the location of the most commonly used points (called ‘interactive ear’) are provided: by selecting auricular points from an ear image on Moodle platform, students can access short narrated videos showing the application of the seed to the point and its main uses.

After the e-learning, there is a five-hour in-person practical class, with case discussions, clinical reasoning exercises, and the selection of points and application of seeds to colleagues. More details on the structure, content, and delivery of the course can be found in Tesser et al.^13,16^

As the course progressed, there was a demand to open it up to some specialized SUS services that deal with problems similar to those in PHC, such as mental health, physical rehabilitation, home care, etc., to which, in agreement with the Brazilian Ministry of Health, the course was extended.

## METHODS

Since its inception, the course has had 45,195 applications, 31,489 approved applications and 20,746 certified graduates as of November 2024. Up until the research period, there had been nine editions of the course, offered annually or biannually, each edition with many classes of approximately 50 to 100 students, distributed in approximately 50 cities across the country to facilitate access to practical classes. The course is not restricted geographically; it is open and free to any healthcare professional with a university degree working in Brazilian PHC (or in Psychosocial Care or Rehabilitation Centers) who seeks training in auriculotherapy, whose responsibility is to attend the practical in-person sessions at their own expense, at the location chosen at the time of registration. These professionals work in Brazilian PHC in various cities and regions across the country.

A cross-sectional observational study was conducted, inviting all graduates from the course to complete an online questionnaire developed by the researchers. The inclusion criterion was to be a graduate from the course, having completed it (there were no exclusion criteria). A first invitation was sent by email to all 13,581 course graduates by March 22, 2023 (with a weekly reminder for three weeks) to complete the electronic questionnaire on the Google Forms digital platform.

The questionnaire was a synthetic adaptation of another one used in a 2017 survey aimed at all course participants at the time, whose main results can be found in Tesser et al.^17^, and which also included questions relating to participant evaluation of the modules, video classes, in-person sessions etc. (which were not of interest at the time of this research and were excluded).

The questionnaire contained 18 questions and an estimated response time of 10 minutes (tested by the researchers themselves—there was no pilot test of the questions, the vast majority of which were identical to those used in the previous survey in 2017^17^). The first set of questions covered sociodemographic data and the professional’s contact with ICHP prior to the course. In order to minimize response time, only six variables were chosen: gender, age, profession, type of service in which they work, previous ICHP experience, and whether they had a ICHP course during their undergraduate studies.

The second set of questions asked about working with auriculotherapy and the perception of its clinical outcomes by practicing professionals. In the same logic as the previous set, only seven variables were collected: whether or not they carry out auriculotherapy after the course; if not, why; the volume of users they see each week; the environment in which they use auriculotherapy (individual consultation, collective care, etc.); whether they record the use of auriculotherapy; user acceptance; and the professionals’ perception of the clinical outcomes obtained with auriculotherapy.

Regarding these outcomes, the focus was on the perception of the effects of auriculotherapy in relation to the objectives for which it was used (which was called ‘perceived effectiveness’ or ‘effectiveness perception’) with the understanding that effectiveness is the clinical outcome obtained in social and institutional life with the therapeutic interventions, i.e., it is the effect of the treatment in the real world of clinical care practice. Efficacy, on the other hand, is the effect of the treatment under ideal conditions, with the patient under strict control and receiving the treatment as it should be applied, such as in a scientifically controlled research conditions of randomized clinical trials (RCTs)^18^. The aim was not to investigate effectiveness in itself, but rather the professionals’ perception of the clinical effects of auriculotherapy, taken here as a proxy for its effectiveness. Even considering this precariousness, investigating this perception is relevant because, despite the greater value and reliability of RCTs for evaluating the effectiveness of treatments, much of medical science is based on observational studies, past practices, and therapeutic tradition^19^. The observation by professionals of the clinical outcomes obtained (despite their biases) directly feeds these last two components, and indirectly the first, by generating hypotheses and motivating new research.

The data obtained from the answers to the questionnaires was analyzed using descriptive statistics (percentages) and in the light of research and scientific evidence on the subject.

The study was approved by the UFSC Human Research Ethics Committee (Opinion 2.345.818 - CAAE: 70289517.4.0000.0121) and the participants digitally signed the Free and Informed Consent Form before accessing the questionnaire, in accordance with Resolution 466/2012.

## RESULTS

A total of 5,461 graduates responded to the survey, 41.34% of those invited. The vast majority of respondents were women (87.82%). The ages of most of the respondents ranged from 30 to 49 years (77.7%). They were nurses (33.66%), followed by physical therapists (11.59%), psychologists (9.65%), pharmacists (8.05%), nutritionists (7.57%), dentists (5.57%), physicians (5.40%), occupational therapists (3.80%), physical education professionals (3.62%), social workers (3.49%), and speech therapists (3.23%); 80.44% worked in PHC services, 7.87% in secondary care, and 5.15% in management. The majority had no previous contact with ICHP during their undergraduate studies (79.22%), only 6.83% had a course on ICHP, and they had no previous experience with any ICHP (73.58%) (Table 1).

**Table 1:** Profile of participating graduates.

	Frequency	Percentage
Gender		
Women	4,796	87.82
Men	646	11.83
Not reported	19	0.35
Total	5,461	100
Age		
20–29	401	7.34
30–39	2,356	43.14
40–49	1,873	34.30
50–59	635	11.63
60–69	166	3.04
70–79	7	0.10
Not reported	23	0.13
Total	5,461	100
Profession		
Nursing	1,832	33.66
Physical therapy	631	11.59
Psychology	525	9.65
Pharmacy	438	8.05
Nutrition	412	7.57
Dentistry	303	5.57
Medicine	294	5.40
Occupational Therapy	207	3.80
Physical Education	197	3.62
Social Work	190	3.49
Speech therapy	176	3.23
Other	65	1.19
Not reported	191	3.18
Total	5,461	100
Area of employment		
Primary care	4,393	80.77
Secondary care	430	7.91
Management	281	5.17
Other activities	335	6.16
Not reported	22	0.40
Total valid types	5,461	100
Learned about ICHP during undergraduate studies		
No	4,326	79.22
Yes, as informal content	725	13.28
Yes, as a formal course	373	6.83
Not reported	37	0.68
Total	5,461	100
Practiced any ICHP before		
No	4,018	73,58
Yes	1,432	26.22
Not reported	11	0.20
Total	5,461	100

ICHP = integrative and complementary health practices.

Note: no question was compulsory.

Regarding their work after the course, 56.31% of the respondents said they practiced auriculotherapy in the SUS. Of those who didn’t practice, almost 80% reported three main types of reasons (they could tick more than one from a list): almost half (46.45%) reported a lack of support from management (lack of supplies, time, opportunity to practice, “management doesn’t make it feasible”); almost a quarter (24.01%) were not practicing (leave, dismissal, management); and 8.39% were in the planning stages of practice. The practitioners’ insecurity (6.12%) and the users’ low acceptance (0.16%) accounted for less than 7% of the reasons for not practicing. Of the practitioners, 16.80% saw more than 15 users a week, 39.03% between 6 and 15 users a week, and 44.17% less than 6 users a week. The environment of use was individual care in 56.56% of the cases, 20.69% in collective care, and 22.75% was auriculotherapy with coworkers. One third (33.13%) said they didn’t record the practice, while 26.97% only recorded it in municipal medical records and 39.90% in e-SUS (federal electronic medical records) with the appropriate code. Of the practitioners who responded about user acceptance, 4.26% reported low or very low acceptance, 22.14% reported moderate acceptance, and 73.6% reported high (44.31%) or very high (29.29%) acceptance. The perceived effectiveness of auriculotherapy was reported as very high or high by 79.72% of respondents, moderate by 18.35% of respondents and low or very low by 1.93% of respondents. Regarding the acceptance of the auriculotherapy practice by the immediate manager over time (last question), 52.54% reported an improvement in the acceptance (Table 2).

**Table 2:** Auriculotherapy practice, acceptance, and perceived effectiveness.

	Frequency	Percentage
Provides auriculotherapy after the course		
No	2,372	43.44
Yes	3,075	56.31
Not reported	14	0.26
Total	5,461	100
If not, why don’t you practice? (more than one answer allowed)		
I am not in primary health care (leave/exoneration/management)	604	24.01
I do not have any supplies	459	18.25
I do not have the opportunity to practice	353	14.04
In planning	211	8.39
I do not have time	179	7.12
Management does not make it possible	177	7.04
I feel insecure about practicing	154	6.12
Users do not accept	4	0.16
Others	374	14.87
Total (of answers)	2,515	100
Auriculotherapy volume in users/week		
More than 15	562	16.80
Between 6 and 15	1,306	39.03
Less than 5	1,478	44.17
Subtotal (practitioners)	3,346	100
Not reported	380	6.70
Not practiced	1,735	31.77
Grand total	5,461	100
Environment of use		
Collective care	881	20.69
Individual care	2,409	56.56
Coworkers	969	22.75
Partial total (practitioners)	4,259	100
Not reported	1,202	22.01
Grand total	5.461	100
Record of use (more than one answer allowed)		
No record	1,479	33.13
Yes, in medical records	1,204	26.97
Yes, recorded in e-SUS	1,781	39.90
Partial total (practitioners)	4,464	100
Not reported	997	18.26
Grand total	5,461	100
User acceptance		
Very high	1,295	29.29
High	1,959	44.31
Moderate	979	22.14
Low	144	3.26
Very low	44	1.00
Partial total (practitioners)	4,421	100
Not reported	652	11.94
Not applicable	388	7.11
Grand total	5,461	100
Perceived effectiveness		
Very high	1,075	24.11
High	2,479	55.61
Moderate	818	18.35
Low	65	1.46
Very low	21	0.47
Partial total (practitioners)	4,458	100
Not reported	615	11.26
Not applicable	388	7.10
Grand total	5,461	100
Has there been a change in the acceptance of the local manager?		
Changed for the better	2,869	52.54
Changed for the worse	55	1.01
No change	1,532	28.05
I took the course recently	168	3.08
Not reported	837	15.33
Grand total	5,461	100

e-SUS: Electronic Medical Record of the Brazilian Ministry of Health.

Note: no answer was mandatory.

## DISCUSSION

The results provide information on a significant proportion (41.34%) of the graduates, who probably played a leading role in popularizing auriculotherapy in the SUS, which was previously little known. Given the magnitude and originality of this course, few studies or experiences are comparable.

A great deal of training in auriculotherapy, albeit smaller than the one discussed here, took place among the US military, who disseminated a pain relief protocol known as ‘battlefield acupuncture’ among their health professionals, involving in-person teaching of 4 to 8 hours for 2,712 professionals in 70 different locations^20^. However, the institutional context of teaching and practice, the disparate use and the very different content prevent comparisons.

A similar survey of participants in the same auriculotherapy course was conducted in the late 2017^17^, via a questionnaire sent by email, which was answered by 57% (n = 2,982) of those invited. The instrument collected evaluations on the different parts of the course and on the graduates’ auriculotherapy work. The profile of the 2017 participants was practically the same as the current one: a large majority of women, nurses in similar proportions, followed by the same other professions, same age profile, same lack of prior learning and practice of ICHP, etc.

In this study, nurses made up a third of the participants, followed by professionals from the multi-professional team (eMulti), the latter in similar proportions to physicians and dentists. In PHC, physical therapists and nurses are the most frequent practitioners of auriculotherapy, followed by nurses, both followed by the other eMulti professions, with physicians in eighth place (dentists do not appear among the ten professions that most frequently perform auriculotherapy)^12^. Since physicians and dentists are more likely to work in PHC than eMulti professionals, it can be deduced that they seek out the course proportionally less, perhaps due to various factors, among which we hypothesize: they are more prejudiced, resistant and/or undervalue auriculotherapy, due to their more rigid biomedical training; perhaps they cannot get authorization to take the course, like other professions; perhaps they think they will not have time to practice. The fact that eMulti is not directly accessed by users may allow them greater flexibility in their schedules and greater interest in new techniques, including those applicable in group settings, such as auriculotherapy. Collective activities are generally more in demand from eMulti, and less so from physicians and dentists, who are pressured more towards conventional individual care. For their part, nurses probably value various care techniques and ICHP more, due to their training and professional tradition.

In the 2017 survey^17^, 72.6% of the participants said they practiced auriculotherapy after the course. This percentage was reduced to 56.31% in 2023, with a persistent report of lack of support from managers as the most frequent reason for not practicing (almost 50% of responses). Although more than half of the respondents reported an improvement in managers’ acceptance of auriculotherapy (a question not included in 2017), this lack of managerial support corroborates a certain invisibility, disregard, and/or undervaluation—within professional and institutional culture—of the contribution of ICHP and auriculotherapy^21,22^. Although staff turnover and attrition may partly explain this finding, it is suggested that state and federal managers encourage municipal managers to support the practice of auriculotherapy, which is simple, low-cost and quick to apply.

The application environments and the volume of users receiving auriculotherapy were also similar, and their under-recording fell slightly, from 36.55% in 2017 to 33.13%. As it is not known whether what professionals record in municipal medical records (26.97%) is passed on to the SUS information system, the real under-recording is probably higher.

The wide acceptance of auriculotherapy by users was similar to that of 2017, although the response options have changed. An explanatory hypothesis for the high acceptance comes from repeated informal reports from graduates: there seems to be rapid word-of-mouth promoted by users who try auriculotherapy, generating high acceptance.

The effectiveness perceived by professionals was also similar to that of 2017, which is in line with the scientific evidence available on the efficacy of auriculotherapy. To support the development of evidence-based clinical auriculotherapy recommendations, produced as complementary material for the course covered, 31 published systematic reviews were analyzed for nine common clinical conditions in PHC (smoking, insomnia, low back pain, obesity, constipation, nausea and vomiting, depression, chronic pain, and rhinitis), of which 8 were of high quality, 19 of acceptable quality and four of low quality, according to the Scottish Intercollegiate Guidelines Network, version 2019 (SIGN-50)^23^, which follows GRADE (Grading of Recommendations, Assessment, Development and Evaluations)^24^. All the reviews suggested that auriculotherapy was more effective than controls, albeit with provisional and parsimonious conclusions, calling for new larger, longer, and better quality clinical trials—this analysis and the respective references can be found on the website of the aforementioned auriculotherapy course^25^.

In the construction of these recommendations, systematic reviews were also produced involving 109 clinical trials evaluating the effectiveness of auriculotherapy for 13 common conditions in PHC: the above-mentioned nine plus anxiety, dysmenorrhea, osteoarthritis, and dental problems. The evaluation of effects also showed positive efficacy compared to controls for all conditions. However, the quality was low in most of the trials: 60% of low quality, 33.7% of acceptable quality, and 6% of high quality, according to SIGN-50. The number of trials for each condition was small, with few patients followed for a short time^25^. Therefore, the positive results deserve caution in their interpretation.

Even so, this evidence must be weighed against the great safety of auriculotherapy^26^and its low cost; its complementary nature; the context of clinical care in PHC, which amplifies safety due to easy access, longitudinality, and the biomedical expertise present there; the great potential of its effect, due to the low severity of most illnesses (greater potential for improvement by self-regulating); the positive experience of practitioners; and, finally, the great acceptance of users. This comparison made it possible to establish positive clinical recommendations for its use in PHC, in accordance with the three pillars of evidence-based care^27^ (critical evaluation of the best available evidence, the experience of professionals and the values and preferences of patients, contextualized in the clinical and institutional circumstances of care)^28^.

In qualitative research with users treated with ICHP invited at random in PHC services, it was observed that in 3/4 of the interviewees (n = 20) this treatment was the initiative of the professionals, in 1/3 of these cases such treatment was proposed as prior to conventional care—leaving drugs as a last resort in case of worsening or failure of ICHP, which was preferred by half of the interviewees, because it is less iatrogenic, more ‘natural’ and stimulates improvement from the ‘inside out’^28^. This suggests that some ICHP, including auriculotherapy, could play a much greater role in PHC than they currently do. Brazilian experiences show that auriculotherapy has been used to welcome users in clinical care and collective activities in PHC and in mental health services^29,30^.

It is recommended that academic managers in the health field expand the teaching of ICHP, including auriculotherapy, in a manner that is careful with its principles and methods, guided by comprehensive care and listening to individuals. The dissemination of auriculotherapy teaching in undergraduate courses could reduce the need for continuing education.

The limitations of this research involve potential selection bias: it cannot be guaranteed that the participants represent the universe of graduates. There may also have been induction bias in the responses. However, the convergence of the two studies discussed and with the data from the SUS information systems suggests that the results are robust. Another convergence is the growing Brazilian literature on auriculotherapy. A search on Google Scholar with the expression “*auriculoterapia atenção primária à saúde SUS*” (“auriculotherapy primary health care SUS”), on January 25, 2025, at 11:22 p.m., generated approximately 8,450 results, of which 5,970 (70%) are from 2016 onwards, when the course began to be offered and auriculotherapy increased in the SUS.

## FINAL CONSIDERATIONS

This study sought to access the profile of the graduates from the auriculotherapy course at UFSC and their work with this ICHP. Most of the participants were women aged 30 to 49 years (77.7%), nurses (33.66%), or professionals from multidisciplinary or family health teams, working in PHC services, with no previous contact with ICHP during their undergraduate studies, and who did not practice ICHP. Of the graduates, 56.31% practiced auriculotherapy after the course, with great acceptance by users and a perception of good clinical results from auriculotherapy. This perception is in line with the scientific evidence available on its efficacy and safety.

These results suggest the desirability of teaching auriculotherapy to health professionals, by expanding the current continuing education strategy, possibly decentralizing/multiplying the offer of the course, and including its teaching in undergraduate courses, training professionals who are already practitioners.

Auriculotherapy has become the most common complementary practice in the SUS, even though it is largely under-recorded and the proportion of graduates practicing it is decreasing, which suggests that it is establishing itself as a care resource in Brazilian PHC. Further research of greater depth and scope is needed to better investigate the insertion of auriculotherapy in the SUS and its contributions to the care provided there.
