# Supplementary material for: Auriculotherapy in primary care: profile of graduates from a large-scale course
Source: Rev Saude Publica. 2025 Oct 24;59:e37. doi: 10.11606/s1518-8787.2025059006715 (PMC12666784; doi:10.11606/s1518-8787.2025059006715)
Supplement: Translation

# Translation: Auriculoterapia na atenção primária: perfil dos egressos de um curso de larga escala

**Authors:** Lúcio José Botelho, Charles Dalcanale Tesser, Melissa Costa Santos, Emiliana Domingues Cunha da Silva, Ari Ojeda Ocampo Moré, Fátima Terezinha Pelachini Farias, Lilian Elizabeth Diesel

**Affiliations:** I Brasil Universidade Federal de Santa Catarina. Centro de Ciências da Saúde. Departamento de Saúde Pública. Florianópolis, SC, Brasil; II Brasil Prefeitura Municipal de Florianópolis. Secretaria de Saúde de Florianópolis. Florianópolis, SC, Brasil

**Keywords:** Terapias Complementares, Auriculoterapia, Atenção Primária à Saúde, Educação Permanente

## Abstract

**OBJETIVOS:**

Apresentar o perfil dos egressos de um curso semipresencial de auriculoterapia e sua percepção da aceitação dos usuários e dos resultados clínicos dessa prática integrativa e complementar em saúde, a mais realizada atualmente no Sistema Único de Saúde. O curso é ofertado gratuitamente aos profissionais da atenção primária à saúde.

**MÉTODO:**

Um questionário digital foi enviado em 2023 por correio eletrônico a todos os egressos até então (n = 13.851), com questões sobre seu perfil profissional, sua percepção da aceitação pelos usuários e dos resultados clínicos da auriculoterapia. Os dados foram discutidos por meio de estatística descritiva.

**RESULTADOS:**

5.461 profissionais responderam (41,34% dos egressos de nove edições do curso, com muitas turmas de cerca de 50 a 100 estudantes). A maioria era composta por mulheres (87,82%), enfermeiros (33,55%), fisioterapeutas (11,59%), psicólogos (5,40%), farmacêuticos (9,65%), nutricionistas (8,05%), dentistas (7,57%) e médicos (5,57%); de idades entre 30 e 49 anos (77,7%); atuantes na atenção primária à saúde (80,44%); sem prática prévia de prática integrativa e complementar em saúde (73,58%). 56,31% reportaram praticar a auriculoterapia após o curso. A aceitação dos usuários foi relatada como alta ou muito alta por 73,6% e média por 22,14% dos praticantes. Os resultados clínicos foram relatados como muito bons ou bons por 79,72% dos praticantes e médios por 18,35%.

**CONCLUSÃO:**

Os egressos do curso de auriculoterapia que responderam à pesquisa são na maioria mulheres, profissionais da saúde da família e das equipes multiprofissionais atuantes na atenção primária à saúde, que relatam boa aceitação dos usuários e percebem bons resultados clínicos no uso da auriculoterapia.

## INTRODUÇÃO

Há algumas décadas, inverteu-se na discussão científica e nos sistemas de saúde a tendência anterior de desconsideração das práticas de cuidado à saúde não científicas (tradicionais ou importadas)^1^, chamadas, entre outras designações, de medicinas tradicionais, complementares e integrativas^2^. Desde a década de 1990, foi reconhecida sua consistente procura por grandes frações populacionais nos países de alta renda^3^, com crescente inserção nos sistemas de saúde^2^ e aumento das pesquisas científicas sobre o tema^4^.

No Brasil, em 2006, foi lançada a Política Nacional de Práticas Integrativas e Complementares (PNPIC)^5^. Desde então, a oferta das práticas integrativas e complementares em saúde (PICS) vem crescendo, sobretudo na atenção primária à saúde (APS)^6^. Em 2017 e 2018, a diversidade de PICS no Sistema Único de Saúde (SUS) foi ampliada para 29 modalidades^7,8^.

O uso clínico das PICS é predominante no setor privado brasileiro^9^. Apesar de seu crescimento no SUS e da diretriz da PNPIC de educação permanente sobre o tema, esta última é ainda incipiente, e poucas iniciativas capacitam os profissionais para a prática clínica de alguma PICS. Uma dessas iniciativas é o curso semipresencial de auriculoterapia, financiado pelo Ministério da Saúde (MS) e realizado pela Universidade Federal de Santa Catarina (UFSC).

A auriculoterapia é uma técnica de cuidado complementar associada à medicina tradicional chinesa (MTC) que consiste em estímulos físicos pontuais no pavilhão auricular para tratamento de problemas de saúde, usando-se mais comumente agulhas (acupuntura auricular) ou sementes vegetais esféricas aderidas à pele, sendo este o modo ensinado no curso citado. Há significativo desenvolvimento de modelos científicos de mecanismos de ação da auriculoterapia^10^, embasando a plausibilidade de sua eficácia.

O curso referido tem sido considerado responsável pelo crescimento da auriculoterapia na APS brasileira^11^, tornando-a a PICS mais ofertada no SUS^12^. É clara a associação (quase linear) do aumento do número de egressos do curso com o aumento da produção de sessões de auriculoterapia na APS, observado nos sistemas de informação do SUS (Gráfico).

**Gráfico:** Egressos do curso e sessões de auricultoerapia na atenção primária à saúde.

Esse curso é a maior iniciativa mundial de capacitação de profissionais convencionais em uma terapia complementar na APS^13^. Foram capacitados, até o final de 2024, aproximadamente 20.000 profissionais do SUS atuantes em quase metade (46%) dos municípios brasileiros (Figura).

**Figura:** Municípios onde atuavam os egressos à época do curso.

Estudos locais e evidências científicas sugerem grande aceitação da auriculoterapia por profissionais e usuários para problemas de alta prevalência e relevância na APS, como problemas de saúde mental^14^ e dores musculoesqueléticas, sobretudo as crônicas, que atingem aproximadamente 30% da população^15^.

Este artigo busca apresentar o perfil dos egressos do curso, sua atuação com auriculoterapia e sua percepção da aceitação pelos usuários e dos resultados clínicos dessa técnica, além de discutir a potencialidade de seu uso clínico disseminado entre profissionais na APS. Antes do método e dos resultados da pesquisa, o curso é brevemente descrito.

### Sobre o Curso

A iniciativa de criação do curso de auriculoterapia partiu do MS, que o encomendou à UFSC em final de 2014. Em 2015, foi elaborado o material didático por uma equipe multidisciplinar, da UFSC e da Secretaria Municipal de Saúde de Florianópolis, que tinha experiência prévia com cursos presenciais de auriculoterapia.

A estruturação do curso foi desenvolvida com base nas diretrizes da Organização Mundial da Saúde (OMS) para a formação em MTC, mas sem aderir à padronização da nomenclatura da acupuntura auricular e ao mapa auricular, padronizados internacionalmente. Em função do caráter introdutório e da necessidade de uma abordagem didática adaptada à APS, o curso integrou os três principais racionais da auriculoterapia contemporânea: a MTC, a reflexologia auricular e os mecanismos neurofisiológicos conhecidos cientificamente. O modelo pedagógico criado incorporou os pontos auriculares mais utilizados na prática clínica e buscou uma abordagem acessível fundamentada tanto na literatura científica como nos referenciais tradicionais da MTC^13^.

Um piloto do curso foi realizado em 2016, cujo material audiovisual sofreu severas críticas e foi reformatado para o formato de videoaulas gravadas em início 2017. Desde então, vem sendo ofertado em sucessivas edições aos profissionais de nível superior da APS.

O curso é composto de cinco módulos de ensino à distância (EaD) sequenciais e autoinstrucionais instalados na plataforma digital *Moodle*, cada um com uma apostila e duas a três videoaulas, previstos para serem cursados em seis semanas: 1) introdução às PICS; 2) reflexologia da orelha; 3) auriculoterapia na lógica da MTC; 4) auriculoterapia à luz da biomedicina; e 5) uso da auriculoterapia na APS. Ao final de cada módulo, o estudante faz uma avaliação e, se aprovado, passa ao módulo seguinte (pode repetir a avaliação o quanto necessário até ser aprovado).

Além das apostilas e videoaulas, são fornecidos um mapa de pontos auriculares (digital e impresso plastificado) e um recurso *online* para estudo da localização dos pontos mais usados, chamado ‘orelha interativa’: ao selecionar pontos auriculares de uma imagem de orelha no *Moodle*, acessa-se pequenos vídeos narrados mostrando a aplicação da semente no ponto e seus principais usos.

Após o EaD, há uma aula prática presencial de cinco horas, com discussões de casos, exercícios de raciocínio clínico e seleção de pontos e aplicação de sementes nos colegas. Mais detalhes sobre a estrutura, o conteúdo e a oferta do curso estão em Tesser et al.^13,16^

Com o andamento do curso, surgiu a demanda para sua abertura para alguns serviços especializados do SUS que atendem problemas similares aos da APS, como de saúde mental, de reabilitação física, de atendimento domiciliar etc., para os quais, em concordância com o MS, o curso foi estendido.

## MÉTODOS

Desde seu início, o curso teve 45.195 solicitações de inscrições, 31.489 inscrições homologadas e 20.746 egressos certificados até novembro de 2024. Até o período da pesquisa, haviam sido realizadas nove edições do curso, oferecidas anualmente ou semestralmente, cada edição com muitas turmas de aproximadamente 50 a 100 alunos, distribuídas em aproximadamente 50 cidades do país, para facilitação do acesso às aulas práticas. A oferta do curso não é restrita geograficamente; ele é aberto e gratuito a qualquer profissional de saúde de nível superior atuante na APS brasileira (ou em Centros de Atenção Psicossocial ou de Reabilitação) que busca a capacitação em auriculoterapia, cujo ônus é responsabilizar-se por comparecer às suas próprias custas ao local da aula prática presencial, escolhido no ato da inscrição. Esses profissionais atuam na APS brasileira em variadas cidades e regiões do país.

Realizou-se um estudo observacional transversal, convidando a totalidade dos egressos do curso, com a aplicação de um questionário *online*, desenvolvido pelos pesquisadores. O critério de inclusão foi ser egresso do curso, tendo-o concluído (não houve critérios de exclusão). Foi enviado por e-mail, um primeiro convite para todos os 13.581 egressos do curso até 22 de março 2023, com um reforço semanal por três semanas, para responderem ao questionário eletrônico na plataforma digital *Google Forms*.

O questionário foi uma adaptação sintética de outro usado em pesquisa de 2017, dirigida a todos os participantes do curso à época, cujos principais resultados estão em Tesser et al.^17^, e que incluía questões relativas também à avaliação pelos participantes dos módulos, videoaulas, parte presencial etc. (que não eram de interesse no momento desta pesquisa e foram eliminadas).

O questionário conteve 18 questões e tempo estimado de resposta de 10 minutos (testado pelos próprios pesquisadores – não houve teste piloto das questões, a grande maioria idênticas às usadas na pesquisa anterior de 2017^17^). O primeiro bloco de questões abordou dados sociodemográficos e o contato do profissional com PICS anterior ao curso. Para minimizar o tempo de resposta, optou-se por apenas seis variáveis: gênero, idade, profissão, tipo de serviço em que atua, prática prévia de PICS e se teve disciplina de PICS na graduação.

O segundo bloco de questões questionou sobre a atuação com auriculoterapia e a percepção dos seus resultados clínicos pelos profissionais praticantes. Na mesma lógica do bloco anterior, apenas sete variáveis foram colhidas: se realiza auriculoterapia após o curso ou não; se não, por quê; qual volume de usuários é atendido por semana; ambiente de uso da auriculoterapia (consulta individual, atividade coletiva, etc.); se registra o uso da auriculoterapia; aceitação pelos usuários; e percepção dos profissionais sobre os resultados clínicos obtidos com a auriculoterapia.

Quanto a esses resultados, focou-se na percepção dos efeitos da auriculoterapia em relação aos objetivos para os quais foi usada, que foi chamada de ‘percepção da efetividade’ ou ‘efetividade percebida’, entendendo-se que a efetividade é o resultado clínico obtido na vida social e institucional com as intervenções terapêuticas, ou seja, é o efeito do tratamento no mundo real da prática do cuidado clínico. Já a eficácia é o efeito do tratamento em condições ideais, com o paciente sob rigoroso controle e recebendo o tratamento como ele deve ser aplicado, como nas condições cientificamente controladas de pesquisa dos ensaios clínicos randomizados^18^. Não se pretendeu investigar a efetividade em si mesma, mas a percepção dos profissionais dos efeitos clínicos da auriculoterapia, aqui tomada como um *proxy* de sua efetividade. Mesmo considerada essa precariedade, investigar essa percepção é relevante, pois, apesar da maior valorização e confiabilidade dos ensaios clínicos randomizados para avaliação da eficácia de tratamentos, grande parte da ciência médica é baseada em estudos observacionais, práticas passadas e tradição terapêutica^19^, e a observação pelos profissionais dos resultados clínicos obtidos (apesar de seus vieses) alimenta diretamente esses dois últimos componentes, e indiretamente o primeiro, ao gerar hipóteses e motivar novas pesquisas.

Os dados obtidos das respostas aos questionários foram analisados por meio de estatística descritiva (porcentagens) e à luz de pesquisas e evidências científicas sobre o tema.

O estudo foi aprovado pelo Comitê de Ética em Pesquisa em seres humanos da UFSC (Parecer 2.345.818 – CAAE: 70289517.4.0000.0121) e os participantes assinaram digitalmente o Termo de Consentimento Livre e Esclarecido anteriormente ao seu acesso ao questionário, conforme a Resolução 466/2012.

## RESULTADOS

Responderam à pesquisa 5.461 egressos, 41,34% dos convidados. A ampla maioria dos respondentes foi de mulheres (87,82%) e, em respeito a essa grande maioria, doravante trataremos o conjunto desses participantes como do gênero feminino, violando as regras da norma culta da língua portuguesa. As idades da maioria das respondentes variaram de 30 e 49 anos (77,7%). Eram enfermeiras (33,66%), seguidas de fisioterapeutas (11,59%), psicólogas (9,65%), farmacêuticas (8,05%), nutricionistas (7,57%), dentistas (5,57%), médicas (5,40%), terapeutas ocupacionais (3,80%), profissionais de educação física (3,62%), assistentes sociais (3,49%) e fonoaudiólogas (3,23%); 80,44% atuantes em serviços de APS, 7,87% na atenção secundária e 5,15% na gestão. A maioria não tinha contato prévio com PICS na graduação (79,22%), apenas 6,83% tiveram uma disciplina sobre PICS, e não praticavam previamente alguma PICS (73,58%) (Tabela 1).

**Tabela 1:** Perfil dos egressos participantes.

	Frequência	Percentual
Gênero		
Feminino	4.796	87,82
Masculino	646	11,83
Não informado	19	0,35
Total	5.461	100
Idade		
20–29	401	7,34
30–39	2.356	43,14
40–49	1.873	34,30
50–59	635	11,63
60–69	166	3,04
70–79	7	0,10
Não informado	23	0,13
Total	5.461	100
Profissão		
Enfermagem	1.832	33,66
Fisioterapia	631	11,59
Psicologia	525	9,65
Farmácia	438	8,05
Nutrição	412	7,57
Odontologia	303	5,57
Medicina	294	5,40
Terapia Ocupacional	207	3,80
Educação Física	197	3,62
Serviço Social	190	3,49
Fonoaudiologia	176	3,23
Outros	65	1,19
Não informado	191	3,18
Total	5.461	100
Serviço em que atua		
Atenção primária	4.393	80,77
Atenção secundária	430	7,91
Gestão	281	5,17
Outras atuações	335	6,16
Não informado	22	0,40
Total de tipos válidos	5.461	100
Aprendeu sobre PICS na graduação		
Não	4.326	79,22
Sim, como conteúdo informal	725	13,28
Sim, como disciplina	373	6,83
Não informado	37	0,68
Total	5.461	100
Praticava alguma PICS antes		
Não	4.018	73,58
Sim	1.432	26,22
Não informado	11	0,20
Total	5.461	100

PICS = práticas integrativas e complementares em saúde.

Nota: nenhuma questão era obrigatória.

Quanto à atuação após o curso, 56,31% das respondentes afirmaram praticar auriculoterapia no SUS. Das que não praticavam, quase 80% reportaram três tipos principais de motivos (podiam assinalar mais de um de uma lista): quase metade (46,45%) referiu falta de apoio da gestão (falta de insumos, de tempo, de oportunidade para praticar, “a gestão não viabiliza”); quase um quarto (24,01%) estava sem atuação clínica (licença, exoneração, gestão); e 8,39% estavam nos estágios de planejamento da prática. A insegurança das profissionais (6,12%) e a pouca aceitação dos usuários (0,16%) foram menos de 7% das razões para não praticar. Das praticantes, 16,80% atendiam mais de 15 usuários por semana; 39,03% entre 6 e 15 usuários por semana e 44,17% menos de 6 usuários semanalmente. O ambiente de uso foi o atendimento individual em 56,56% dos casos, 20,69% em atividades coletivas e, em 22,75%, era auriculoterapia com colegas de trabalho. Um terço (33,13%) afirmou não registrar a prática, enquanto 26,97% registravam apenas em prontuário municipal e 39,90% no e-SUS (prontuário eletrônico federal) com código adequado. Das praticantes que responderam sobre aceitação dos usuários, 4,26% relataram baixa ou muito baixa aceitação, 22,14% reportaram média aceitação e 73,6% assinalaram alta (44,31%) ou muito alta (29,29%) aceitação. A efetividade percebida da auriculoterapia foi relatada como muito alta ou alta por 79,72% das respostas; média em 18,35% das respostas e baixa ou muito baixa em 1,93% das respostas afirmativas, considerando as que responderam à questão. Sobre a aceitação da prática da auriculoterapia pelo gestor imediato ao longo do tempo (última pergunta), 52,54% relataram melhoria dessa aceitação (Tabela 2).

**Tabela 2:** Prática da auriculoterapia, aceitação e efetividade percebida.

	Frequência	Percentual
Faz auriculoterapia após o curso		
Não	2.372	43,44
Sim	3.075	56,31
Não informado	14	0,26
Total	5.461	100
Se não, por que não pratica? (mais de uma resposta permitida)		
Não estou na atenção primária à saúde (licença/exoneração/gestão)	604	24,01
Não tenho insumos	459	18,25
Não tenho oportunidade para praticar	353	14,04
Em planejamento	211	8,39
Não tenho tempo	179	7,12
Gestão não viabiliza	177	7,04
Sinto-me inseguro para praticar	154	6,12
Os usuários não aceitam	4	0,16
Outros	374	14,87
Total (de respostas)	2.515	100
Volume de auriculoterapia em usuários/semana		
Mais de 15	562	16,80
Entre 6 e 15	1.306	39,03
Menos de 5	1.478	44,17
Subtotal (praticantes)	3.346	100
Não informado	380	6,70
Não pratico	1.735	31,77
Total geral	5.461	100
Ambiente de uso		
Atendimento coletivo	881	20,69
Atendimento individual	2.409	56,56
Colegas de trabalho	969	22,75
Total parcial (praticantes)	4.259	100
Não informado	1.202	22,01
Total geral	5.461	100
Registro do uso (mais de uma resposta permitida)		
Não registro	1.479	33,13
Sim, no prontuário	1.204	26,97
Sim, registro no eSUS	1.781	39,90
Total parcial (praticantes)	4.464	100
Não informado	997	18,26
Total geral	5.461	100
Aceitação dos usuários		
Muito alta	1.295	29,29
Alta	1.959	44,31
Média	979	22,14
Baixa	144	3,26
Muito baixa	44	1,00
Total parcial (praticantes)	4.421	100
Não informado	652	11,94
Não se aplica	388	7,11
Total geral	5.461	100
Efetividade percebida		
Muito alta	1.075	24,11
Alta	2.479	55,61
Média	818	18,35
Baixa	65	1,46
Muito baixa	21	0,47
Total parcial (praticantes)	4.458	100
Não informado	615	11,26
Não se aplica	388	7,10
Total geral	5.461	100
Houve mudança na aceitação do gestor local?		
Mudou para melhor	2.869	52,54
Mudou para pior	55	1,01
Não mudou	1.532	28,05
Fiz o curso recentemente	168	3,08
Não informado	837	15,33
Total geral	5.461	100

eSUS: Prontuário Eletrônico do Ministério da Saúde.

Nota: nenhuma resposta era obrigatória.

## DISCUSSÃO

Os resultados trazem informações sobre uma parte significativa (41,34%) dos egressos, os quais provavelmente protagonizaram a popularização da auriculoterapia no SUS, antes pouco conhecida. Dada a magnitude e a originalidade desse curso, poucos estudos ou experiências são comparáveis.

Uma grande capacitação em auriculoterapia, porém menor do que a tratada aqui, ocorreu entre os militares norte-americanos, que disseminaram entre seus profissionais de saúde um protocolo para alívio da dor conhecido como *battlefield acupuncture*, abrangendo ensino presencial de 4 a 8 horas para 2.712 profissionais em 70 locais diferentes^20^. Todavia, o contexto institucional de ensino e prática, o uso díspar e o conteúdo muito diverso impedem comparações.

Pesquisa similar com participantes do mesmo curso de auriculoterapia foi realizada no final de 2017^17^, via questionário enviado por correio eletrônico, respondida por 57% (n = 2.982) dos convidados. O instrumento coletava avaliações sobre as diferentes partes do curso e sobre a atuação com auriculoterapia dos egressos. O perfil dos participantes de 2017 foi praticamente igual ao atual: grande maioria de mulheres, enfermeiras em proporções similares, seguidas pelas mesmas outras profissões, mesmo perfil etário, mesmas ausências de aprendizado e de prática prévia de PICS etc.

Na presente pesquisa, as enfermeiras constituíram um terço das participantes, seguidas dos profissionais da equipe multiprofissional (eMulti), estes em similar proporção que médicas e dentistas. Na APS, os fisioterapeutas e enfermeiros são os maiores praticantes de auriculoterapia, seguidos dos enfermeiros, ambos seguidos das demais profissões das eMulti, com os médicos em oitavo lugar (dentistas não aparecem entre as dez profissões que mais realizam auriculoterapia)^12^. Como médicos e dentistas estão em maior número na APS que profissionais das eMulti, deduz-se que procuram proporcionalmente menos o curso, talvez por vários fatores, dentre os quais hipotetizamos: têm maior preconceito, resistência e/ou subvalorizam a auriculoterapia, devido à formação biomédica mais rígida; talvez não consigam autorização para fazer o curso, como as demais profissões; talvez considerem que não terão tempo para praticar. O fato das eMulti não serem diretamente acessadas pelos usuários talvez lhes possibilite uma maior flexibilidade de agenda e maior interesse por novas técnicas, incluindo as aplicáveis em ambientes grupais, como a auriculoterapia. Atividades coletivas são geralmente mais demandadas das eMulti, e menos de médicos e dentistas, pressionados mais para cuidado convencional individual. Por seu turno, enfermeiras, provavelmente, valorizam mais diversas técnicas de cuidado e PICS, pela sua formação e tradição profissional.

Na pesquisa de 2017^17^, 72,6% das participantes afirmaram praticar auriculoterapia após o curso. Essa porcentagem foi reduzida para 56,31% em 2023, com persistência do relato de falta de apoio dos gestores como o motivo mais frequente para não praticar (quase 50% das respostas). Apesar de mais da metade das respondentes relatarem melhoria na aceitação da auriculoterapia pelos gestores (questão inexistente em 2017), essa falta de apoio dos gestores corrobora certa invisibilidade, desprezo e/ou subvalorização na cultura profissional e institucional da contribuição das PICS e da auriculoterapia^21,22^. Embora a rotatividade e evasão de profissionais possam explicar parte desse achado, sugere-se que gestores estaduais e federal estimulem os gestores municipais a apoiarem a prática da auriculoterapia, simples, de baixo custo e rápida de aplicar.

Os ambientes de aplicação e o volume de usuários que recebem auriculoterapia também foram similares, e seu subregistro reduziu pouco, de 36,55% em 2017 para 33,13%. Como não se sabe se o que o profissional registra em prontuário municipal (26,97%) é repassado ao sistema de informações do SUS, o subregistro real provavelmente é maior.

A grande aceitação da auriculoterapia pelos usuários foi semelhante à de 2017, embora as opções de resposta tenham sofrido alteração. Uma hipótese explicativa para a grande aceitação vem de repetidos relatos informais de egressos: parece haver uma rápida propaganda boca-a-boca promovida pelos usuários que experimentam a auriculoterapia, gerando grande aceitação.

A efetividade percebida pelos profissionais também foi similar à de 2017, o que converge com as evidências científicas disponíveis sobre eficácia da auriculoterapia. Para subsidiar a elaboração de recomendações clínicas de auriculoterapia baseadas em evidências, produzidas como material complementar do curso abordado, foram analisadas 31 revisões sistemáticas publicadas para nove condições clínicas comuns na APS (tabagismo, insônia, lombalgia, obesidade, constipação, náusea e vômito, depressão, dor crônica e rinite), das quais oito eram de alta qualidade, 19 de qualidade aceitável e quatro de baixa qualidade, conforme o Scottish Intercollegiate Guidelines Network, versão 2019 (SIGN-50)^23^, que segue o GRADE (Grading of Recommendations, Assessment, Development and Evaluations)^24^. Todas as revisões sugeriam haver eficácia da auriculoterapia superior aos controles, ainda que com conclusões provisórias e parcimoniosas, demandando novos ensaios clínicos maiores, mais longos e de melhor qualidade – essa análise e as referências respectivas estão no *site* do referido curso de auriculoterapia^25^.

Na construção dessas recomendações, também foram produzidas revisões sistemáticas envolvendo 109 ensaios clínicos que avaliavam a eficácia da auriculoterapia para 13 condições comuns na APS: as nove acima citadas mais ansiedade, dismenorreia, osteoartrite e problemas odontológicos. A avaliação dos efeitos também mostrou haver eficácia positiva em relação aos controles para todas as condições. Todavia, a qualidade foi baixa na maioria dos ensaios: 60% de baixa qualidade, 33,7% de qualidade aceitável e 6% de alta qualidade, conforme o SIGN-50. Foi pequeno o número de ensaios para cada condição, com poucos pacientes seguidos por pouco tempo^25^. Logo, os resultados positivos merecem cautela na sua interpretação.

Mesmo assim, essas evidências devem ser confrontadas com a grande segurança da auriculoterapia^26^ e seu baixo custo; seu caráter de prática complementar; o contexto do cuidado clínico na APS, que amplifica a segurança devido ao acesso fácil, à longitudinaldiade e à expertise biomédica ali presente; a grande potencialidade de seu efeito, devido à baixa gravidade da maioria dos adoecimentos (maior potencial de melhora por autorregulação); a experiência positiva dos praticantes; e, por fim, a grande aceitação dos usuários. Essa confrontação permitiu estabelecer, conforme os três pilares do cuidado baseado em evidências^27^(avaliação crítica das melhores evidências disponíveis, experiência dos profissionais, valores e preferências dos pacientes, contextualizados nas circunstâncias clínicas e institucionais do cuidado), recomendações clínicas positivas para seu uso na APS^28^.

Em pesquisa qualitativa com usuárias tratadas com PICS convidadas aleatoriamente em serviços da APS, observou-se que em 3/4 das entrevistadas (n = 20) esse tratamento foi iniciativa dos profissionais, em 1/3 desses casos tal tratamento foi proposto como anterior ao cuidado convencional – deixando fármacos para caso de piora ou insucesso das PICS, o que foi preferido por metade das entrevistadas, por ser menos iatrogênico, mais ‘natural’ e estimular melhora de ‘dentro para fora’^28^. Isso sugere que algumas PICS, incluída a auriculoterapia, poderiam ter um papel na APS bem maior do que o atual. Experiências brasileiras mostram que a auriculoterapia vem sendo usada no acolhimento aos usuários, em atendimentos clínicos e atividades coletivas na APS e serviços de saúde mental^29,30^.

Recomenda-se aos gestores acadêmicos da área da saúde a ampliação do ensino das PICS, incluindo a auriculoterapia, de modo cuidadoso para com seus princípios e métodos, orientado pela integralidade do cuidado e a escuta dos sujeitos. A disseminação do ensino da auriculoterapia nas graduações poderia reduzir a necessidade de educação permanente.

Os limites desta pesquisa envolvem potencial viés de seleção: não se pode garantir que as participantes representam o universo das egressas. Pode também ter havido viés de indução nas respostas. Todavia, a convergência das duas pesquisas discutidas e com os dados dos sistemas de informações do SUS sugere robustez dos resultados. Outra convergência é a crescente literatura brasileira sobre auriculoterapia. Busca no Google Scholar com a expressão “auriculoterapia atenção primária à saúde SUS”, em 25 de janeiro de 2025, às 23h22min, gerou aproximadamente 8.450 resultados, dos quais 5.970 (70%) são de 2016 em diante, quando iniciou a oferta do curso e o aumento da auriculoterapia no SUS.

## CONSIDERAÇÕES FINAIS

Este estudo buscou acessar o perfil dos egressos do curso de auriculoterapia da UFSC e de sua atuação com essa PICS. A maioria das participantes foram mulheres de 30 e 49 anos (77,7%), enfermeiras (33,66%) ou profissionais da equipe multidisciplinar ou da saúde da família, atuantes em serviços de APS, sem contato prévio com PICS na graduação, não praticantes de PICS. Das egressas, 56,31% praticavam auriculoterapia após o curso, com grande aceitação pelos usuários e percepção de bons resultados clínicos da auriculoterapia. Tal percepção converge com as evidências científicas disponíveis sobre sua eficácia e segurança.

Esses resultados sugerem a desejabilidade do ensino da auriculoterapia aos profissionais de saúde, via ampliação da atual estratégia de educação permanente, possivelmente descentralizando/multiplicando a oferta do curso, e da inserção de seu ensino nas graduações, formando profissionais já praticantes.

A auriculoterapia se tornou a prática complementar mais realizada no SUS, mesmo com grande subregistro e uma diminuição da proporção de egressos praticantes, sugerindo que ela está se estabelecendo como um recurso de cuidado na APS brasileira. Novas pesquisas de maior fôlego e abrangência são necessárias para melhor investigar a inserção da auriculoterapia no SUS e suas contribuições para o cuidado ali realizado.
